# A logic framework for addressing medical racism in academic medicine: an analysis of qualitative data

**DOI:** 10.1186/s12910-024-01045-9

**Published:** 2024-04-15

**Authors:** Pamela Roach, Shannon M. Ruzycki, Kirstie C. Lithgow, Chanda R. McFadden, Adrian Chikwanha, Jayna Holroyd-Leduc, Cheryl Barnabe

**Affiliations:** 1 Department of Family Medicine, Cumming School of Medicine, University of Calgary, Calgary, Canada; 2 Department of Medicine, Cumming School of Medicine, University of Calgary, 3330 Hospital Drive NW, Calgary, AB 1422, T2N 2T9 Canada; 3 Department of Community Health Sciences, Cumming School of Medicine, University of Calgary, Calgary, Canada; 4 Department of Allied Health, Alberta Health Services, Calgary, Canada; 5 Department of Cardiac Sciences, Cumming School of Medicine, University of Calgary, Calgary, Canada

**Keywords:** Racism in medicine, Equity, diversity, and inclusion, Healthcare workforce

## Abstract

**Background:**

Despite decades of anti-racism and equity, diversity, and inclusion (EDI) interventions in academic medicine, medical racism continues to harm patients and healthcare providers. We sought to deeply explore experiences and beliefs about medical racism among academic clinicians to understand the drivers of persistent medical racism and to inform intervention design.

**Methods:**

We interviewed academically-affiliated clinicians with any racial identity from the Departments of Family Medicine, Cardiac Sciences, Emergency Medicine, and Medicine to understand their experiences and perceptions of medical racism. We performed thematic content analysis of semi-structured interview data to understand the barriers and facilitators of ongoing medical racism. Based on participant narratives, we developed a logic framework that demonstrates the necessary steps in the process of addressing racism using if/then logic. This framework was then applied to all narratives and the barriers to addressing medical racism were aligned with each step in the logic framework. Proposed interventions, as suggested by participants or study team members and/or identified in the literature, were matched to these identified barriers to addressing racism.

**Results:**

Participant narratives of their experiences of medical racism demonstrated multiple barriers to addressing racism, such as a perceived lack of empathy from white colleagues. Few potential facilitators to addressing racism were also identified, including shared language to understand racism. The logic framework suggested that addressing racism requires individuals to understand, recognize, name, and confront medical racism.

**Conclusions:**

Organizations can use this logic framework to understand their local context and select targeted anti-racism or EDI interventions. Theory-informed approaches to medical racism may be more effective than interventions that do not address local barriers or facilitators for persistent medical racism.

**Supplementary Information:**

The online version contains supplementary material available at 10.1186/s12910-024-01045-9.

## Background

Racism that disadvantages patients and physicians has been well-documented in peer reviewed literature [1, 2], government reports [3, 4], and the media [5–7], in Canada [8], the United States [9], and abroad [10]. While anti-racism may seem to have recently emerged in commentaries and in institutional statements [11], regular calls [3, 4, 12] for physicians to address racism have been made since at least the early 1990s [13]. Despite this, racism in the medical field has persisted and continues to be misunderstood [6] or denied [14], causing harm to physicians [15, 16] and patients [8, 17]. 

A cross-sectional survey of physicians in Alberta to explore racism found that Black, Indigenous, and People of Colour (BIPOC) physicians experience a higher prevalence of racism in the workplace than their white peers [18]. Analysis of text responses in this survey and others [15, 19] provide some understanding of the ways that racism manifests in healthcare, its impact on physicians and patients, and proposes possible interventions to address racism; [20–22] however, qualitative analysis of interview data that deeply explore experiences and beliefs about racism in medicine are less common [23]. Further, there is not a unifying, multi-level framework to understand drivers of persistent racism in the medical workplace to inform intervention design. The aim of this current study was to explore physician experiences and perceptions of racism in a Canadian university using semi-structured interviews to inform a model of drivers of persistent racism.

### Terminology

Race is a social construct without biologic meaning that is used to categorize people into groups based on their appearance, which are then assigned societal value [24]. Racial discrimination is disadvantaging a person based on their perceived race, and racism is racial discrimination plus use of power to alter outcomes for a group of people [24]. White people may experience racial discrimination but not racism, due to their privilege in society [24, 25]. 

In this study, we grouped participant racial identities into white or Black or Indigenous, Asian, and People of Colour (POC) to avoid potential identification of participants from distinct categories. This categorization is meant to differentiate participants who can experience racism (BIPOC) and those who cannot (white); however, these categories are heterogenous and arbitrary. Many groups feel that the term BIPOC enforces hierarchies among people of colour [26]. We use this term in this manuscript after discussion with study team members with lived experience of racism and colonization, despite these limitations.

### Setting

Alberta has a single universal healthcare system serving 4.4 million people. There are approximately 11,000 practicing physicians of which 59% are male and 41% are female [27]. Survey data suggests that less than 3% of practicing physicians in Alberta are gender diverse, transgender, non-binary gender, or Two-Spirit [28]. Based on survey and census data, an estimated 3–5% of Alberta physicians are Black, 1–3% are Indigenous, 1–3% are Latinx/Hispanic, 5% are Middle Eastern, 10% are South Asian, 7% are East Asian, and 50–70% are white [28]. Explicit and implicit anti-Black [6] and anti-Indigenous [29] interpersonal racism have been documented in Alberta [30], and this racism results in differential health care delivery for racialized groups [1, 31]. 

## Methods

This qualitative interview study was approved by the University of Calgary Conjoint Health Research Ethics Board (REB20-1688) and is reported according to the Consolidated criteria for Reporting Qualitative Research (COREQ) guidelines [32]. 

### Participants

All clinical members (physicians, residents, and nurse practitioners) in the Departments of Medicine (*n* = 420), Family Medicine (academic members only; *n* = 40), Cardiac Sciences (*n* = 125), and Emergency Medicine (*n* = 220) in the Cumming School of Medicine, University of Calgary (*n* = 774) were invited to be interviewed about racism in the workplace via a single e-mail from department leadership. These departments were selected because each has an EDI (equity, diversity, and inclusion) committee that can act on the results of this study to implement solutions. All eligible participants were interviewed, without restriction or purposeful sampling by race, with an ethical imperative that all interested participants be offered the opportunity to take part. Further, inclusion of perspectives from racialized and white participants was necessary to address the study objective of understanding experiences and perceptions of medical racism. A single invitation was sent to reduce the burden of e-mails and tasks during the COVID-19 pandemic. Saturation was assessed by inductive thematic saturation, which considers the appearance of new codes or themes rather than the development of existing themes [33]. Participation was voluntary and compensated with a $50 gift card. All participants provided informed consent, including consent for use of quotes in knowledge dissemination materials.

### Interviews

The interview guide was developed based on the study questions and a review of the literature (Appendix 1). Because the aim of this project was to explore physician’s experiences of racism in medicine broadly, the interview guide was to allow participants to guide the interview based on their motivations and priorities. The proposed interview guide was circulated to various university EDI committees for feedback prior to use; it is possible that study participants may have provided input into the developed interview guide. Semi-structured interviews were conducted virtually between April and August 2021 due to local COVID-19 pandemic protocols. Each interview was audio-recorded and transcribed verbatim. Interviewers de-identified transcripts prior to analysis. Participants reviewed their transcripts to suggest edits at their own discretion.

### Analysis

Thematic content analysis [34] was guided by constructivism, which allows the existence of multiple truths and realities [35], and performed in NVivo (version 12.3.0, QSR International, Inc., Doncaster, Australia). Initial codes were developed deductively using Dr Camara Jones’ Levels of Racism framework [36], which organizes racism to three levels: institutional, interpersonal, and internalized. Additional codes were generated inductively through close reading of all transcripts by S.M.R. and C.R.M. Coding was completed independently, in parallel, by S.M.R. and C.R.M. with three transcripts to generate a codebook. The codebook was presented to the entire study team with exemplar quotations to assess validity. The final codebook was then applied to all transcripts (Appendix 2). Each transcript was independently analyzed by two study team members with training in qualitative data analysis (S.M.R. and K.C.L. or C.R.M.) and disagreements were reconciled through discussion with the wider study team.

Themes were developed by examining the most prevalent codes for relationships, patterns, commonalities, and differences between participants. After the initial analysis, it was clear that most participant descriptions of an experience of racism had repetitive, ordered elements; a description, an internal reaction, a rationalization, and an external reaction. We therefore organized each narrative about an experience of racism into ordered components to create a logic framework for addressing racism. Logic frameworks are often used to define the purpose and activities of a program by organizing the steps required to achieve the overall project goal [37]. In our logic framework, the goal was addressing racism in the medical workplace, where ‘addressing’ could mean any personal or organizational intervention to mitigate racism. We then attempted to identify the barrier or facilitator of addressing racism for each narrative by asking “What allowed (or prevented) the participant (or institution) from addressing racism in this experience?”. Each narrative was re-examined in this model and barriers or facilitators at each step were tabulated and consolidated into major categories (Appendix 3).

To hypothesize solutions related to each barrier or facilitator, we searched peer-reviewed literature and consulted with colleagues with expertise in EDI. Consultation with experts was solicited by e-mail and through formal discussion during EDI committee meetings in the participating departments.

### Reflexivity

Participants were able to select from four trained interviewers (C.R.M., P.R., A.N.C., and S.M.R.), who are diverse in racial identity, gender, profession, and department affiliation. C.R.M. is a white cisgender woman social worker and P.R. is a Métis woman and primary researcher. A.N.C. is a Black man physician. S.M.R. is a white cisgender woman physician. C.R.M., S.M.R., and K.C.L. participated in data analysis. K.C.L. is a white cisgender woman physician. The remaining members of the study team, who provided interim feedback and validation of the study design, data collection and data analysis, also included Métis (C.B) and white (J.H.L.) cisgender women physicians.

### Funding

This study was funded internally by the Cumming School of Medicine’s Department of Medicine Vice Chair for Indigenous Health, held by one of the study authors (C.B.).

## Results

### Overview

Nineteen interviews (17–90 min) were completed, with respondents representing all participating departments (2.5% participation rate). Respondents were diverse in race and gender identity (Table 1). Saturation was reached in creation of the logic framework after 12–15 interviews, as no additional codes or themes were developed after this point. Experiences of or witnessed medical racism were shared by all but one (white) participant. Participant reactions to explicit interpersonal racism varied; while some participants felt reassured knowing that “there was a reason for his (behaviour), it’s not a rational reason, but at least there seems to be an explanation” (BIPOC Participant (BP)1), others felt that “every time it happens, it’s a bit like a slap in the face” (BP7). Many were hesitant to attribute these experiences to racism. Instead, they offered other potential explanations, including “ignorance” (BP4), “genuine surprise” (BP2), “curiosity” (BP16), and “laziness” (BP3).

**Table 1:** Demographic data of interview participants

Characteristic	Number of Participants n (%)
Total participants	19
Gender identity	
Cisgender women	11 (58%)
Cisgender men	8 (42%)
Transgender, gender diverse, non-binary gender or Two Spirit	0
Racial identity	
White	5 (26%)
BIPOC^*^	14 (74%)
Intersecting gender identity and race	
BIPOC cisgender women	8 (42%)
BIPOC cisgender men	6 (32%)
White cisgender women	4 (21%)
White cisgender men	1 (5%)

^*^BIPOC refers to Black, Indigenous and People of Colour to describe a heterogenous group of people who can experience racism. We have combined this extremely diverse group to protect individual identities, especially when there are few people in our setting who identify with certain groups. In this study, this group included Black, Egyptian, Persian, Indian, Indigenous, Hispanic, and Asian participants and participants who identified as multiple racial or ethnic groups. Several of these participants also identified with a religious group that experiences marginalization

### Addressing racism by addressing barriers and leveraging facilitators: an anti-racism logic framework

**Fig. 1:**
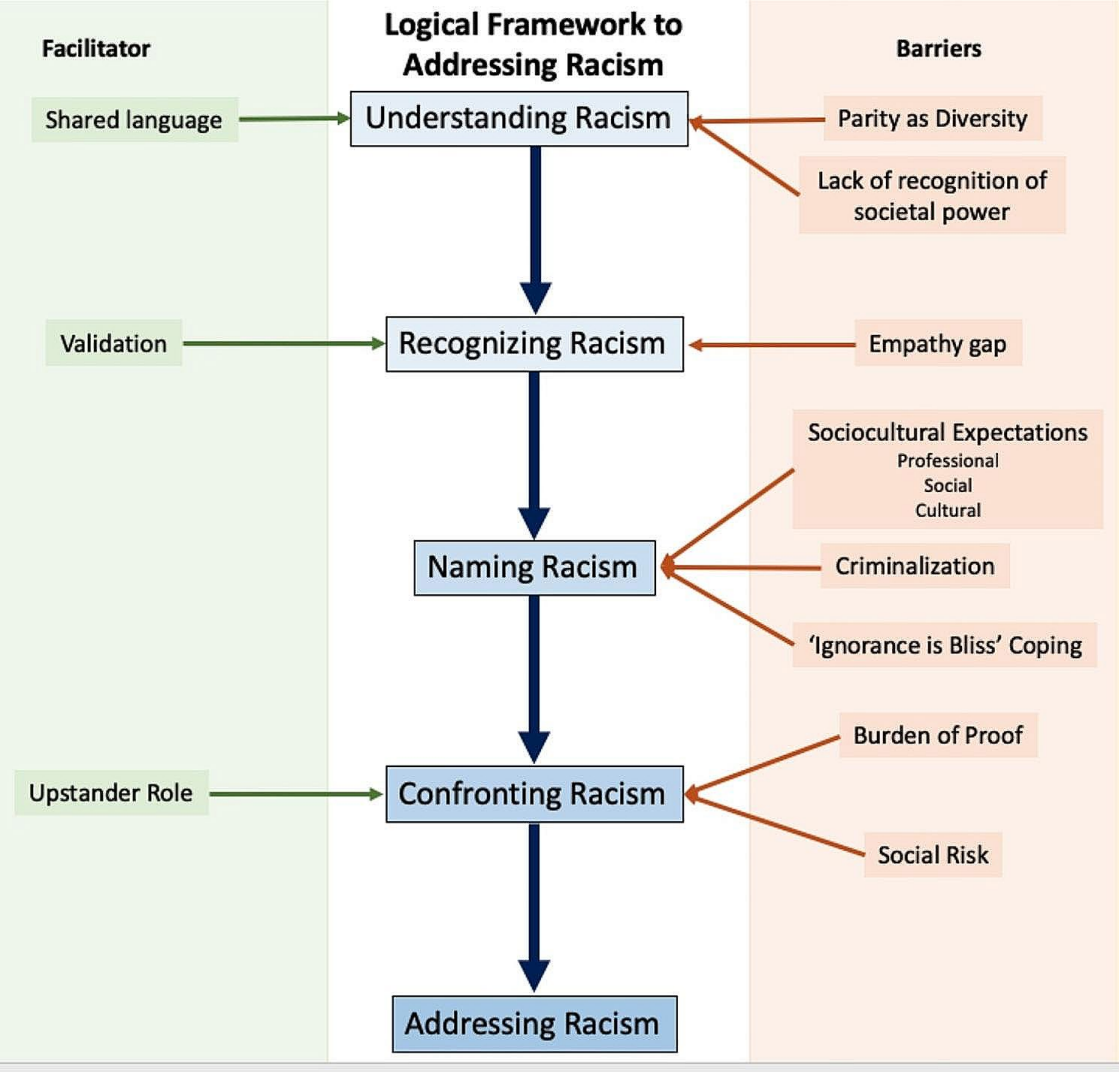
Logic framework derived from qualitative analysis of interview data. There is a cascading sequential process to addressing racism requiring understanding, recognizing, naming and confronting racism

The logic framework included understanding, recognizing, naming, and confronting racism as processes required to address racism (Table 2; Fig. 1). Participant narratives illustrated how each step in this model interacted to prevent addressing racism; for example, if participants did not understand racism, they were unable to recognize racism when it occurred, and participants who did not name an experience as racism were unable to confront racism. This framework allowed us to identify barriers to addressing racism that could be targeted by interventions. Our analysis is presented here in detail.

**Table 2:** Constructs of the logic framework for address racism in medicine with an explanation of their logic and the accompanying facilitators of racism based on participant responses

Construct	Explanation	Facilitators of Racism
Understanding racism	The participant expresses an understanding of racism^*^ (or lack of racism) that is incomplete or incorrect.	• Conflating the presence of BIPOC physicians with a lack of racism • Attributing a perceived lack of malicious intent by the perpetrator as an absence of racism • Lack of understanding of how systems of power are required to convert racial bias to racism
Recognizing racism	A participant who understands racism is unable to attribute racism as a factor in an episode or interaction that they experienced or witnessed.	• The invisibility of privilege^†^ for white physicians who benefit from racism • Subtlety of structural and implicit bias [33] • Lack of validation for BIPOC physicians that their experiences differ from white colleagues due to racism
Naming racism	A participant who recognizes that they have experienced racism is unable to use the term “racism” to describe the experience.	• Sociocultural expectations and norms: • Maintenance of the physician-patient relationship • Colourblindness^‡^ • Cultural gratefulness • Coping strategies among BIPOC physicians that focus on denial or ignoring discomfort
Confronting racism	A participant who names that they experienced racism is prevented from confronting the process or person that was racist.	• The burden of proof on the targets of racism to prove ‘beyond a reasonable doubt’ that racism contributed to their experience • The criminalization of racism such that racism is seen as such a strong or offensive accusation that it is rude or mean to use this term [38] • Social risk of those who ‘play the race card’

BIPOC = Black, Indigenous and People of Colour to describe a heterogenous group of people who can experience racism

^*^Racism is racial bias (discrimination or stereotypes directed at a person based on their membership in a racial group) plus power (a privileged^†^ position [36] in society that gives one power to disadvantage another group)

^†^An unearned advantage given to someone by a society or culture based on characteristics such as ability, skin colour, or gender identity [36].

^‡^Colourblind racism is an ideology that purports race is no longer a relevant social category, e.g., “I don’t see skin colour” [39]. This ideology ignores how structural, internalized, and interpersonal racism continue to disadvantage groups of people

### Understanding racism

Most participants struggled to conceptualize racism. Participants were unsure whether malintent rather than “ignorance or laziness” (BP7) was required. Some participants described a shift in their understanding of intent versus impact throughout their careers and as societal conversations around racism progressed, emphasizing learning about racism as a facilitator for defining racism. Further, most participants did not have a conceptual understanding of racism as being the combination of racial bias and power differential resulting in disadvantages. For example, when asked about experiences of racism, one participant shared that a food service worker told them that a meal had pork in it, and described this as “discrimination, but they’re actually trying to be helpful… because they’re culturally aware that people who look like me don’t eat meat or bacon sometimes,” (BP17). Nearly all participants provided examples of representation as evidence for a lack of racism.

### Recognizing racism

Many BIPOC participants wondered “deep down” (BP9, BP16, BP19) if racism had affected their career trajectory. Some felt that, while they could not point to a particular instance, there may be evidence of subtle effects over their entire career: “I’ve never thought of race having an impact in terms of… career advancement opportunities. But recently, when I think back on it, it makes me wonder if there was some degree of that playing a role,” (BP9) and “I’ve not felt that there’s been any attempt to slow me down due to my race (though) sometimes you think that might be the case because it takes you a little longer to get there than others who may be of other racial backgrounds” (BP16). For some, this wondering occupied significant mental space: “You sort of sit back and say ‘Is it because I’m female? Is it because I’m visible minority?’ And you can’t really piece it out. So, I find that is sometimes challenging,” (BP14). Similarly, the invisibility of white privilege was demonstrated by the multiple white participants who assumed that their race “likely allowed some good luck to come my way” though they “don’t recall any specific instances when that happened,” (white participant (WP) 15). Unlike BIPOC participants, no white participant reported distress from the possibility that race had influenced their career trajectories.

BIPOC participants perceived an empathy gap when their white colleagues did not recognize their experiences of racism. Some white participants were aware of the gap between their recognition of workplace racism and their colleagues’ experiences, stating that their view of the workplace as equitable is “a major assumption for me to be making as not part of a racialized minority group,” (WP18). In this way, safe spaces where experiences were shared and validated as racism were a facilitator to counteract the belief that “I thought [racism] happened to just me, I thought it was just like, normal,” (BP8).

Having a shared language to discuss racism was a facilitator for recognizing racism. One participant shared that “I wasn’t familiar with (the) term (microaggressions) until I went to (a) talk. And since that talk, I’ve heard it talked about in all kinds of places…it really resonated with me,” (BP2).

### Naming racism

Sociocultural expectations, referring to the professional, social, or cultural norms that police behaviour, were a prominent barrier to naming racism. For example, participants were cautious about “causing a ruckus” (BP2), “rocking the boat” (BP4), “ruffling feathers” (BP12), or being a “hassle” (BP3) when “playing the race card” (BP3, BP9) to describe an experience, worrying that it would “skewer my career” (BP8). Participants felt that “it’s a big deal to call someone a racist, it’s almost one of those taboo terminologies that you save for those people that are mobbing and lynching people,” (BP2). This ‘criminalization’ of race and racism contributes to “an unspoken rule… in healthcare and society” to not discuss race and racism (BP12) and left participants without the language to describe their experiences.

Participants shared that they were raised to “be grateful” for their opportunities, to “work twice as hard as everyone else” (BP1, BP8), and not to “complain” or cause problems (BP2, BP4, BP19), often attributing these values to their parents or culture. With patients, participants felt that they need to be “the better person… the professional” to avoid making patients “uncomfortable” (BP1). Several participants shared that they suppressed their feelings or even memories of racist events as a coping mechanism because “if you think about it, you’d be upset all the time,” (BP1).

### Confronting racism

Participants who recognized racism were hesitant to report or confront these experiences because they felt obligated to ‘prove’ their experiences to others. This was especially true for implicit racial bias and systemic discrimination. Participants felt that “it’s a hard case to make, unless you’re very obviously discriminated against and you have a really solid base,” (BP2) and “people within minority groups don’t feel comfortable raising these issues, because then all of a sudden you have to prove (it) to everybody,” (BP9).

Further, participants felt that they risked their reputation by reporting racism, because “people are going to think (I) can’t make it on (my) own merit” (BP2). A shared language to discuss racism facilitated confronting racism; for example, one participant found it helpful to refer to an education session: “I can just say ‘Remember that thing we talked about? You’re doing it right now,’” (WP13). The social risk of confronting racism was overcome when participants witnessed their colleagues as the targets. One participant used the need for trainees “to be in a place where they can feel safe and comfortable learning” (WP6) as justification for dismissing an explicitly racist patient from their practice whereas being the target of racism themselves was not a justification. Some white participants felt cautious speaking up on behalf of their racially marginalized colleagues, “How do you support those people without taking away their agency… but also making sure that they feel safe and they can speak up?” (WP13).

### Anti-racism interventions

We identified barriers and facilitators to addressing racism (Fig. 1), and then matched these to anti-racism interventions that were suggested by participants directly, developed using the study team members’ expertise, and/or based on peer-reviewed literature. These are summarized in Table 3 and are briefly described here.

**Table 3:** Constructs from the logic framework for addressing racism matched to interventions identified in the literature and through discussion with experts that are hypothesized to address the underlying gap in logic

Logic	Intervention	Mechanism	Explanation
Understand racism	EDI Moments	Build literacy and understanding of EDI-related concepts.	Modelled after ‘Safety Minutes’ used in industry to build safety literacy and culture [37], EDI Moments are 5–15 minute presentation on a single EDI-related concept held at the beginning of leadership meetings.
Recognizing racism	Peer Support	Provide opportunity to share experiences and receive validation from trained peers.	Peer support programs use shared lived experience and empathetic listening to validate experiences. In medicine, peer support programs reduce distress from adverse events [40].
	Race & Racism Teaching Rounds	Provide real examples of how structural and interpersonal racism influence patient care.	Modelled after “Morbidity and Mortality Rounds”, Race and Racism Rounds apply a just culture approach to improving patient care by identifying contributors to adverse outcomes [41].
	Story Telling	Share the experiences of physicians from marginalized groups [42–44] to build empathy.	Narrative reflections may demonstrate how racism and other forms of discrimination manifest for people from marginalized groups.^61^
	Implicit Bias Training^62^	Teach physicians to recognize and mitigate the effects of unconscious beliefs.	Implicit Bias Training workshops have increased hiring of underrepresented groups in academic settings.^63^
Naming racism	Local guidelines for patient originating harassment	Address social norms that prevent physicians from naming racism.	Systems-level algorithms for an organizational response to patient requests for white physicians, microaggressions, or other forms of explicit interpersonal harassment shifts from the individual judgements and penalties for action to a collective strategy for these issues.
	Support Networks	Provide a safe space to talk about racism.	Modelled after Women in Medicine groups to create a space for sharing removed of judgement and bias.
	Disclosure training	Develop skills among medical leadership to receive disclosures of harassment and discrimination.^64^	Lack of support or dismissal by leadership when reporting an experience of racism, sexism, harassment, or discrimination is a central barrier to harassment reporting [18].
Confronting racism	Bystander Intervention Training	Build skills to address racism in the moment it occurs.	Confronting racism is a skillset that must be learned and practiced [45].
	Harassment Reporting Mechanisms	Address known barriers to reporting harassment.	Most harassment reporting mechanisms for physicians are not transparent, anonymous, confidential, or safe for those reporting, which contributes to underreporting of harassment among physicians.^65^ Many physicians do not know how to report workplace harassment [18].
	Remediation-Based Approaches to Harassment.	Use a report of racism as an opportunity to improve.	Adopting a ‘just culture’ and/or restorative justice approach (when applicable) to racism allows for improvement and growth rather than defensiveness.

EDI = equity, diversity, and inclusion

### Building knowledge to Understand Racism

Organizations can adapt occupational safety strategies [46] to focus on racism and other EDI concepts. For example, to build collective and organizational knowledge, leaders could reserve time at the beginning of meetings for brief presentations on a single EDI topic [41]. 

### Developing skills in recognizing racism

Physicians can learn how to recognize medical racism through formal Race and Racism Rounds, modelled after Morbidity and Mortality Rounds, where the adverse clinical outcomes attributable to structural and interpersonal racism are made explicit and discussed [42]. Similarly, workplace racism can be shared through formal story telling; in these programs, published narratives from physicians who have experienced racism or discrimination [43, 44, 47] are shared and discussed to build empathy and understanding for all physicians. Lastly, facilitated implicit bias training workshops can build skills to recognize racism [48]. 

### Structural supports for naming racism

Organizations can develop policies that guide decision-making when racism occurs. For example, a directive that establishes the steps that will occur when a patient requests a white physician removes the need for individual physicians to self-advocate. Similarly, creation of safe spaces where physicians who experience racism can network and share their experiences may remove barriers faced by BIPOC physicians when seeking support. Peer support programs can link physicians who experience harassment or discrimination to trained peers with similar lived experiences. All physician leaders must have the skills to appropriately respond to disclosures of racism from their colleagues, to avoid the common experience of being dismissed or unsupported when reporting racism. The number and handling of harassment and discrimination concerns should be regularly reported to organization members to promote accountability [45]. 

### Taking action to confront racism

As barriers to understanding, recognizing, and naming racism are addressed, organizations must create structures to facilitate confrontation of medical racism. Bystander intervention training can provide skills for physicians to leverage their privilege to safely confront racism in real time [49]. Organizations must develop evidence-based harassment reporting mechanisms that address known barriers to reporting [50, 51] and incorporate remediation-based and restorative justice approaches to harassment.

## Discussion

This study of 19 white and BIPOC faculty members in a single medical school identified that most participants struggled to understand, recognize, name, and confront racism due to individual and structural factors. In a logic framework, these barriers worked together to prevent people and systems from addressing racism in the medical workplace. We matched these barriers to proposed interventions to reduce the barriers or amplify facilitators of addressing racism in medicine. Medical leaders may be able to use these results to identify barriers and facilitators most relevant to their context and implement interventions that target these drivers of ongoing racism.

Previous cross-sectional surveys of Black Canadian physicians estimated a prevalence of workplace racism as 71% [15], which is in keeping with the prevalence seen among BIPOC physicians in Alberta and across North America [19, 28] While many of these studies performed qualitative analysis of survey text responses, analysis of interview data may provide important contextual details on how racism can manifest in medicine [34]. Several qualitative studies of Black, Native American, and Hispanic academic faculty [20, 23] physicians [22, 23], and residents [21] reported the ubiquity of racial microaggressions, the requirement to ‘represent’ ones entire race or ethnicity, and social and professional exclusion, including lack of mentorship. Our study builds on the results of these studies by examining participant reflections on racism in addition to describing their prevalence and impact. This allows us to identify barriers to addressing racism and match interventions to each barrier.

Editorials calling on physicians to address racism in medicine are common [52] and physicians must not forget that addressing racism in medicine is an ethical obligation of their profession [4, 53]. Interventions to address racism in medicine that do not target underlying contributors are unlikely to be successful. This may explain the conflicting data on the effectiveness of interventions such as implicit bias training [54] or candidate demographic masking [55, 56], which are unlikely to be effective in settings where implicit bias is not the primary contributor to racism. Conceptual frameworks can help researchers understand and address complex phenomena [57] and have been used to examine persistent sexism in medicine [58, 59]. Our framework has similarities to the adapted Information-Motivation-Behavioural Skills Model developed by Jindal and colleagues to understand how an anti-racism curriculum could address medical racism among pediatric residents [60]. This model helps understand factors that impede or promote behavioural interventions by describing the relationship between information, motivation to change, skills needed to change, action planning, and behaviour change. The motivation and action planning domains observed in Jindal’s model were not identified among our data, though the need for information (e.g., understanding) and skills (e.g., recognizing) to address racism were common to both. This finding may be partially due to differences in the study population; in Jindal et al.’s study, 67% of participants were white compared to 5% of our participants. White participants more often mentioned they are motivated to change, given their “sense of responsibility noting their agency to name racism in the moment” [60] and “one’s own participation in [racist] systems” [60] than BIPOC participants. Further, in contrast to our exploration of participant experiences of racism, Jindal et al. specifically asked participants about how their anti-racism curricula may lead to changes in clinical practice, which likely prompted more responses that related to the action planning domain [60]. 

Our framework provides insights into how institutions can assess where gaps in their current anti-racism work exist along this framework and provides a rationale to select interventions that target these gaps. For example, leaders may use surveys [38] to determine whether their members have an accurate conceptualization of racism (understanding) before attempting to implement bystander intervention training (confronting) [49]. Similarly, institutions may benefit from local guidelines to guide physicians in addressing harassment from patients only after its members can recognize racism and its impact on their colleagues.

There are several limitations to this study. The first is possible selection bias, as this was a study examining a sensitive topic, and so our results may represent only the views of those who are most interested or confident about this topic. Social desirability bias may have influenced participant responses due to the presence of an interviewer. Selection and social desirability bias may be why we did not identify explicit interpersonal racism as an important barrier to addressing racism in this study, though explicit racism exists among physicians [43] and surely plays a role in the persistence of racism in medicine. For this reason, our logic framework may only apply when institutions have addressed explicit racism. Similarly, we did not seek to test the logic model developed from our data and so this is hypothesis-generating only. The logic model should be examined in other settings; it would be important to see if this framework can be used to predict which anti-racism interventions will be most effective. Our results represent the experiences of physicians in different academic departments in a single university and facilitators of racism may be different in settings with different histories or structures of racism and oppression. For example, in settings where anti-racism work is currently being opposed by government and lobby groups [39] or where anti-Mexican bias is a more predominant form of racism than is typically seen in our setting [40], there may be different barriers and facilitators.

## Conclusions

Overall, this thematic analysis of qualitative data builds on cross-sectional prevalence data and other qualitative explorations of participant experiences to describe how physicians in a single medical school perceive racism in their setting. These results informed a logic model that requires individuals to understand, recognize, name, and confront racism before racism can be effectively addressed by individuals and organizations. Interventions targeted to gaps in this logic model may be better positioned to tackle the challenging and persistence of racism in medicine that harms patients, trainees, and physicians.

### Electronic supplementary material

Below is the link to the electronic supplementary material.

Supplementary Material 1

## Data Availability

Select data may be available upon reasonable request from the corresponding author; however, due to the sensitivity of the study data, the full data set will not be provided.

## Declarations

### Author information

P.R. and C.B. are Métis women who have lived experience of anti-Indigenous racism in the healthcare system. A.C. is a Black man. S.M.R., K.C.L., C.R.M., and J.H.L. are white women.
