# Supplementary material for: A logic framework for addressing medical racism in academic medicine: an analysis of qualitative data
Source: BMC Med Ethics. 2024 Apr 15;25:45. doi: 10.1186/s12910-024-01045-9 (PMC11017534; doi:10.1186/s12910-024-01045-9)
Supplement: Supplementary file 1 — Supplementary Material 1 [file 12910_2024_1045_MOESM1_ESM.docx]

A Logical Framework for Addressing Racism in Medicine: Analysis of Qualitative Data

Appendices

| Appendix 1. Interview guide | 2 |
| --- | --- |
| Appendix 2. Final Codebook | 5 |
| Appendix 3. Illustration of the logic framework using participant narratives. | 9 |

**Appendix 1**. Interview Guide.

Introduction

Review consent form – Have they signed it? Do they have questions? Do not start interview before participant consents.

Have they completed the optional pre-interview demographic survey? We are using these data to ensure that we have recruited a diverse group of participants prior to data analysis. It can be completed after the interview as well.

Emphasize that you are recording their responses and that you will de-identify them before they are shared with the study team. Let them know that their information can be removed at any point by contacting you, up until the moment of publication. Please tell them that you will circulate all published or publicly available materials using their data before they are disseminated for them to approve. Please emphasize that their participation is completely confidential; only you will know that they participated in this project.

Race is a social construct with no biological equivalent or distinction between groups. All groups are “racialized”, including white people. There are many controversies in how to refer to individuals who are minoritized or disadvantaged in our society due to race. In this study, we will use the term “BIPOC”, or Black, Indigenous and People of Colour, to refer to the group of people who are disadvantaged, marginalized and/or minoritized due to racism in Canada. Individuals who experience discrimination due to ethnicity, referring to shared culture or heritage (e.g., Jewish or Hispanic people) are included in this definition. We acknowledge the limitations of this term. If you prefer to use another term, please let us know.

Some of the questions or discussion may be traumatizing. The interview can be stopped at any time and any question can be skipped. The Physician and Family Support Program, Office of Professionalism, Equity and Diversity, and Homewood Health programs are available to all participants.

Interview Guide

1. Can you describe yourself and your role as a physician for context? (Prompt – FFS/AMHSP/GFT, roles, teaching, clinical, specialty, etc).
2. Which racial group(s) do you identify with? (*Skip to white faculty questions if they respond ‘white’*).

BIPOC faculty

1. Do you think other people perceive you to be [from the racial group as described by the participant]? (*If not, have you thought of your experience being different than those from your population group(s) that are visibly non-white?*)
2. Can you describe the culture of your department when it comes to racial equity? Do you feel that this culture is inclusive and equitable (or not)?
3. Do you feel your race impacted your career trajectory in your department? (*If so, how/If not why not*?)
4. What have your experiences been as a [use self-described term from Question 2] physician? Do you feel that you have been treated differently than White colleagues? Who treated you differently (e.g. leadership, administration staff, colleagues, trainees, patients)? What was your response to these experiences? How do others react when you share these experiences with them? Who is your support for when these events happen?
5. How do these experiences differ between your clinical, academic, research, and education roles?
6. Have you experienced discrimination or harassment due to other aspects of your identity, such as sex, gender, or ability? (*If yes - How do you think race has influenced these experiences? Do you think a White physician with this characteristic has different or similar experiences?*)
7. What barriers have you experienced as a physician? How have these barriers impacted you? (*Prompt – your career, your specialty choice, your practice location, your mental health or career satisfaction?*) How do you feel that these barriers differ from your White colleagues?
8. What changes would you like to see in the Cumming School of Medicine that would address racism?

White faculty

1. Do you consider yourself to be a visible minority? [If not - Have you thought of your experience being different than those from your population group(s) that are visible?]
2. Do you feel that BIPOC colleagues are treated differently in your department?
3. Can you describe the culture of your department when it comes to racial equity? How do you feel that this culture is inclusive and equitable (or not)?
4. Have you witnessed your BIPOC colleagues being treated differently? [Prompt- in clinical, education, research, administration?] What was your response to these experiences? How did others react when you shared these experiences with them?
5. Have you experienced discrimination or harassment due to other aspects of your identity, such as sex, gender, or ability? (*If yes - How do you think race has influenced these experiences? Do you think a BIPOC physician with this characteristic has different or similar experiences?*)
6. What barriers have you experienced as a physician in your department? How have these barriers impacted you? (*Prompt – your career, your specialty choice, your practice location, your mental health or career satisfaction?*) How do you feel that these barriers differ from your BIPOC colleagues?
7. What changes do you think the Cumming School of Medicine could make to address racism?

**Appendix 2**. Final Codebook.

| **Node** | **Code** | **Definition** | **Details** |
| --- | --- | --- | --- |
| Experiences of Race and Racism | | | |
|  | Attribution/Certainty | Expresses certainty (and uncertainty) about whether an experience was (or was not) racism; attributes an experience to another phenomenon; states they aren't sure if something is racist; declares they cannot know a person's motivations |  |
|  | Colourblindness | Describes their own or another person's belief that all people should be seen the same regardless of race; describes being 'race-blind' or lack of acknowledgement of the different experiences of people from different racial groups. | Type of racism that perpetuates white supremacy |
|  | Culture | Participant describes a cultural or societal contributor (or modifier) to their experience of racism (or lack of experience of racism).  Exclude: Participant describes a cultural experience of their race or religion (e.g., describes how they celebrate Ramadan) | Participant describes something about Canadian or provincial cultural that modified their experiences. |
|  | Definition | Describes a definition of racism; describes how one knows whether an experience was racism or not racism  Include: Classified an event as 'not racist' with explanation of why; | When a participant states that something was not racist due to intent |
|  | Department/Division | Describes a characteristic or experience specific to their Department or Division related to an experience of race or racism (or lack of race/racism) | Would use to understand our current state |
|  | Double standard | When participant feels that they are evaluated or treated or perceived differently than someone of a different race (or was not seen differently) |  |
|  | Internalized racism | Expression of ideas, beliefs, or actions that support or collude with racism.  Includes having a sense of inferiority, expressed victimhood and denying personal agency, feeling overwhelmed/drained by emotions due to navigating race, focus on trying to change white people. Rewards conformity with white people. |  |
|  | Interpersonal racism | Describes an episode of racial discrimination (witnessed or experienced) that occurs between two individuals.  Include: Any race-based discrimination; any participant defined episode without adjudication by study team members; Study team member adjudicated racism (e.g., participant describes an episode of interpersonal racism but does not describe as racism or describes specifically as not racism); participant remarks on lack of interpersonal racism  Exclude: Structural or internalized racism. | Include when a participant says that someone "is not racist". |
|  | Microaggression | Participant describes an experience (or lack of experience) of a microaggression (e.g., "Where are you from" or being mistaken for another health care provider)  Exclude: Interpersonal racism | Can refer to 11 microaggressions article |
|  | Structural racism | Describes an episode of racial discrimination (witnessed or experienced) that is rooted in structures, systems, or policies.  Include: Any race-based structural or systemic discrimination; any participant defined episode without adjudication by study team members; Study team member adjudicated racism (e.g., participant describes an episode of structural racism but does not describe as racism or describes specifically as not racism); participant remarks on lack of interpersonal racism  Exclude: Interpersonal or internalized racism. | Include when a participant says that a system "is not racist" or if participant is unsure whether observed differences are due to race or if participant is unsure whether observed differences are due to race. |
|  | Representation | Participant describes the diversity (or lack of diversity) of a place or describes how seeing (or not seeing) diversity leads to change. |  |
|  | Experiences of Race | Experience related to race that is not attributed as racism by the participant and the interviewer.  Do not include if the participant calls this racism.  Do not include if you feel that it meets the definition of racism |  |
| Identity | | | |
|  | Work demographics | Participant describes the characteristics of their job or role. | To understand who participated, does not have to be related to racism or race. |
|  | Perceived identity | Participant describes how other people perceive their identity, related to race, gender, etc. |  |
|  | Intersectionality | Participant describes how their identities overlap to modify their experiences.  Include: "I am a Muslim woman"; My identity as a woman is more important than being Egyptian"  Exclude: participant lists their identities (code as personal demographics); e.g., "I am a mother, I am Christian" |  |
|  | Modifying characteristics | Describes personal (or family, or other) characteristics that protect them (or someone else) from racism or the consequences of racism; describes characteristics that make them (or someone else) more susceptible to racism or the effects of racism.  Do not include "Intersectional" factors - like also being a woman, etc. |  |
|  | Personal demographics | Participant describes personal characteristics, e.g., age, religious identity, etc. |  |
| Confronting or Report Racism | | | |
|  | Deciding to report | Describe factors involved or the process of deciding whether or not to report racism/harassment/discrimination. |  |
|  | Formal reporting | Describes an episode where they reported an episode of racism/harassment/discrimination. |  |
|  | Deciding to confront | Describe factors involved or the process of deciding whether or not to confront an action of racism/harassment/discrimination.  Exclude: descriptions of formal reporting mechanisms or reporting to leadership. |  |
|  | Confronting | Describes an episode of intervening or confronting when witnessing or experiencing harassment, racism or discrimination.  Do not include formal reporting to leadership/the College, etc. |  |
| Impacts of experiences | | | |
|  | Behaviour change | Changes to behaviours or habits or actions as a result of racism (or lack of change).  Do not include career outcomes. |  |
|  | Career | Changes (or lack of changes) to career or experiences of work or expectations of work or career path due to racism or expected racism.  Include interpersonal racism from decision-makers that the participant feels influenced their selection. |  |
|  | Coping | Strategies for coping with racism (or lack of need to cope with racism). |  |
|  | Safety | Impact of racism on perception of safety (or lack of impact on perception of safety). |  |
|  | Well-being/emotions | Impact (or lack of impact) of racism on feelings or emotions. |  |
| Solutions | | | |
|  | Solutions | Any proposed solution or suggestion to reduce discrimination.  Include: Comments on interventions that have not or would not work. |  |

**Appendix 3.** Participant narrative responses to questions about potential experiences of racism that illustrate how the logic framework helps to understand barriers to addressing racism and the potential mitigating interventions.

| **Narratives** | **Logic / Constructs** - explanation |
| --- | --- |
| Participant:  I remember there was one time, so I was at the Tim Hortons's at the hospital cafeteria. I bought my coffee and **the person who served me the coffee, she looked at me and she's like, "I saw the best movie on the weekend. Kung Fu Panda. Have you seen it?"**  I assume, and maybe this is just my erroneous assumption, **maybe she just thought it was a really amazing movie and she would've mentioned that comment to anyone**, **but I think she mentioned it specifically because I was Asian and probably had seen the movie.**  I've come in with a mindset… "the world works better if you assume people are trying their best." **So I just assumed she just saw me and then thought about the movie she saw and thought maybe it would be interesting**, but I've never gone up to a complete stranger and been like, "I just saw Three Billboards Outside Ebbing, Missouri and it was an amazing movie. Have you seen it?"  I think now it bothers me a lot more, but I don't know how to address that discomfort. **Ten years ago, we weren’t talking about all these things**... I think all those things I just would've let slide, like **I was happy to be in my tunnel and just get my clinical experience and finish my residency… there is a sentiment within the Asian community that you don't rock the boat.**  Interviewer:  When you think back to that Kung Fu Panda thing, at the time, did you really think that she would've said that to a white woman or do you think it was because of your race?  Participant:  **I think it was totally because of my race.** I literally think she was saying it because I was Asian.  So I do think it was targeted, but maybe I'll give her the benefit of doubt, but maybe that's just to make myself feel better… but I don't know. **Again, when I look back, I'm like, it's just ignorance and maybe it's easier to say it's ignorance than racism.** | **Interpersonal racial microaggression**^64^ – an intentional or unintentional comment or behavior that serves to create a hostile environment or communicate disrespect for someone from a marginalized group. |
|  | **Understanding racism** – participant expresses initial uncertainty about whether an incident was racism or not.  **Understanding racism** – participant expresses that initially they did not view the incident as racist because the participant did not have malintent. |
|  |  |
|  | **Shared language** as a facilitator for understanding racism. |
|  | **Cultural expectation** as a barrier to naming racism. |
|  |  |
|  | **Recognizing racism** – participant feels that an incident happened because of their race. |
|  |  |
|  | **Naming racism** – participant is hesitant to call this incident racism due to an ‘ignorance is bliss’ coping strategy. |
| Interviewer:  Is racism or microaggressions common for you at work?  Participant:  I don't think it's necessarily common. **It’s hard to subscribe [something to race]**.  My name is not easy for people to pronounce correctly. **I did notice like our site lead, for quite a few years now, [a decade], [supervising colleague] still can't pronounce my last name correctly**.  Interviewer:  And have you felt like you could correct them?  Participant:  Oh yeah. **I corrected people… just how many times should I correct them?** Because I pronounced it correctly for them before. **It's sometimes difficult. And sometimes it's not worth the hassle** sometimes I feel like.  Interviewer:  What do you mean by hassle?  Participant:  Chinese, it's a tonal language. So **trying to get that tonal language correct is sometimes difficult and then just, it would take a few minutes and probably for them to practice and whatnot. And it might be harder for them to remember too, because growing up in an English language society, there are certain things that are easier to pronounce sometimes**.  **And from a non-ethnic standpoint, names get butchered all the time.** |  |
|  | **Understanding racism** – participant expresses initial uncertainty about whether an incident was racism or not. |
|  | **Interpersonal racial microaggression** – an intentional or unintentional comment or behavior that serves to create a hostile environment or communicate disrespect for someone from a marginalized group. |
|  |  |
|  | **Confronting racism** –the work of addressing this racism is too much of a burden for the participant. |
|  |  |
|  | **Sociocultural expectation** that it is too much work for English-speaking people to learn their name as a barrier for naming racism. |
|  | **Understanding racism –** participant does not recognize this instance as racism because they do not understand the link between social power and racism. |
